# Novel biosensor for high-throughput detection of progesterone receptor-interacting endocrine disruptors

**DOI:** 10.1038/s41598-024-55254-8

**Published:** 2024-03-06

**Authors:** Diana A. Stavreva, Lyuba Varticovski, Razi Raziuddin, Gianluca Pegoraro, R. Louis Schiltz, Gordon L. Hager

**Affiliations:** Laboratory of Receptor Biology and Gene Expression, National Cancer Institute, NIH, 41 Medlars Dr., Bethesda, MD 20892-5055 USA

**Keywords:** Biological activity, Hormones, Screening, River water, Environmental impact, Fluorescent proteins

## Abstract

Progesterone receptor (PR)-interacting compounds in the environment are associated with serious health hazards. However, methods for their detection in environmental samples are cumbersome. We report a sensitive activity-based biosensor for rapid and reliable screening of progesterone receptor (PR)-interacting endocrine disrupting chemicals (EDCs). The biosensor is a cell line which expresses nuclear mCherry-NF1 and a green fluorescent protein (GFP)-tagged chimera of glucocorticoid receptor (GR) N terminus fused to the ligand binding domain (LBD) of PR (GFP-GR-PR). As this LBD is shared by the PRA and PRB, the biosensor reports on the activation of both PR isoforms. This GFP-GR-PR chimera is cytoplasmic in the absence of hormone and translocates rapidly to the nucleus in response to PR agonists or antagonists in concentration- and time-dependent manner. In live cells, presence of nuclear NF1 label eliminates cell fixation and nuclear staining resulting in efficient screening. The assay can be used in screens for novel PR ligands and PR-interacting contaminants in environmental samples. A limited screen of river water samples indicated a widespread, low-level contamination with PR-interacting contaminants in all tested samples.

## Introduction

The Progesterone Receptor (PR) is a central player in the progesterone signaling pathway and regulates expression of many genes involved in development, differentiation and proliferation of target tissues^1^. In addition, PR is implicated in hormonally dependent cancers^1–5^. Environmental contaminants in waste and surface waters containing endocrine disrupting chemicals (EDCs ) which interact with PR may alter reproductive fitness and development of aquatic organisms, and have serious effects on human health^6^.

PR, also known as nuclear receptor subfamily 3, group C, member 3 (NR3C3), is a nuclear receptor (NR) activated by the steroid hormone progesterone (P4). In humans, PR is encoded by the *PGR* gene residing on chromosome 11 (11q22-q23) and it has two isoforms, PR-A (94 kDa), and PR-B (116 kDa)^7,8^. These two isoforms are identical in amino acid sequence, except for a 165 amino acid extension at the N-terminus of the PR-B isoform^9,10^. Although the two isoforms are frequently co-expressed, the additional activation domain in the N-terminus of PR-B confers important functional differences, and changes in the PR-A/PR-B expression ratio are implicated in endometriosis and breast cancer^11–13^. In addition to P4 binding, PR activity depends on developmental stage, tissue/cell-type accessibility of target promoters and enhancers, availability of co-regulatory proteins, and the activity of signaling pathways which regulate posttranslational modifications^4,14–18^.

Compounds that bind PR have different pharmacological properties and mode of action^19,20^. However, natural and synthetic molecules having progestational activity are collectively known as progestins. The sources of progestins in the environment are ubiquitous. Blood levels of the natural P4 hormone peak in the human and other mammals’ female menstrual cycle and are further elevated during pregnancy. In addition to its physiological functions in humans and livestock, P4 is also excreted in urine and milk into the environment. Other environmental sources are synthetic progestins in contraceptives, hormone replacement therapy, and in treatment of endometriosis and several cancers. Many synthetic steroids having androgenic, estrogenic and/or glucocorticoid activities also exert progesterone-like activity^21,22^. Progesterone and its synthetic analogs are consumed in larger amounts than any other sex hormone^21,23^. When excreted, these chemicals and their metabolites contaminate the environment^24^. The concentration of progestins in wastewater and surface water sources ranges from a few to tens ng/L; higher concentrations have been reported (reviewed in^21,24^. For example, androgenic progestins, routinely used as growth promoters in livestock, resulted in 11,900 ng/L in livestock manure and soil, and 350 ng/L in the associated runoffs^25^. In recent studies untreated sewage was a major source of EDCs in China^26^. 29 EDCs, including six progestins were identified in a Chinese lake during an overgrowth of blue-green algae. Many countries have reported environmental progestins in rivers, such as in Hungary Danube River where progesterones were < 0.37 ng/L^27^, USA National scale Rivers 63.0 ng/L^28^, France Rhône-Alpes 1.7–3.5 ng/L^29^, Brazil, 0.51–47.2 ng/L^30^, South Korea Han River < 0.5 ng/L^31^, China, Xiangjiang River, up to 72.2 ng/L ^26^, Blanice River in the Czech Republic, concentrations in the range of 0.82–1.1 ng/L^32^.

Progestins are proposed to undergo over 90% biodegradation in wastewater plants^33–35^. However inefficient progestin removal was reported from the Czech Republic, Slovakia^36^, Malaysia^37^, and Argentina^38^. The progestins Norethindrone and Levonorgestrel, in combination with other steroids, have been identified by liquid or gas chromatography-tandem mass spectrometry (LC–MS/MS and GC–MS/MS) in wastewater treatment plant effluents at concentrations of up to 50 ng/L^29,39^. Medroxyprogesterone, another well-known synthetic progestin^40^ that affects sex differentiation and spermatogenesis in zebrafish^41^ was detected in municipal wastewater plants effluents and surface water samples at the levels up to 15 and 1 ng/L, respectively^42^.

Thus, contamination with progestins alone or in combination with other steroid hormones is largely due to human activities and may occur widely in the environment.

Multiple highly sensitive analytical methods for detection of these EDCs have been developed recently^43,44^. Although these methods are sensitive, they are expensive, time-consuming and for the most part not suitable for high-throughput screening of environmental samples. Furthermore, they only detect known compounds and cannot detect bio-active metabolites of PR ligands, which are not present in the reference chemical structure libraries. Many steroid metabolites found in the environment were reported to retain strong endocrine disrupting effects^23^; however, frequently only the levels of the “parent” steroids are considered. For example, the concentration of progesterone metabolites in wastewater can exceed that of the parent compound^45–47^. Shen et al^48^ reported that the progesterone metabolite 3α-hydroxy-5β-tetrahydroprogesterone in river water was 63 times higher than that of the parent compound. Because the levels of environmental steroid metabolites may be seriously underestimated, assays that are focused on PR biological activity endpoints are potentially better suited at detecting and monitoring steroid contaminants in environmental samples. Finally, a major disadvantage of current screening biological endpoint methods is that detection of antagonists is difficult^49,50^.

Considering that progestogens are important EDCs impacting aquatic biota at very low concentrations, it is important to develop sensitive, high-throughput, effect-based screening methods which provides rapid detection of these contaminants in environmental samples.

To address this unmet need, we developed a rapid, high-throughput imaging, activity-based cellular assay for detection of EDCs with progesterone-like activities and demonstrated its applicability to detect PR-interacting chemicals in contaminated river water samples.

## Materials and methods

### Chemicals

Progesterone (P4), Mifepristone (RU-486), Dexamethasone (Dex), Hydrocortisone (HydroCort), Testosterone (Testo) and the organic solvent DMSO were purchased from Sigma (catalog Nu: P0130-25G, 84,371-65-3, D1756, H4001, T1500-1G, and D2650, respectively). Gestogene (CAS No. 60282-87-3), Norgestimate (CAS No. 35189-28-7), Altrenogest (CAS No. 850-52-2), Chlormadinone Acetate (CAS No. 302-22-7), Norethindrone (CAS No. 68-22-4), Norgestrel (CAS No. 6533-00-2), Levonorgestrel (CAS No. 797-63-7), Etonogestrel (CAS No. 54048-10-1), Asoprisnil (CAS No. 199396-76-4), Ulipristal Acetate (CAS No. 126784-99-4) were purchased from Cayman Chemical.

### Grab water samples collection

River water samples were collected in the summer of 2018 from different geographic locations of Mattaponi River (Table 1). These grab water samples were collected in pre-cleaned glass gars and stored at 4 °C before filtration through a GF/F filter (0.7 µm) using a solvent rinsed all-glass apparatus. We collected two samples from each location in the same instance. They were processed and tested independently and reported as Replica 1 and 2 (R1 and R2). Next, filtered samples and blanks were subjected to solid phase extraction (SPE) using OASIS® HLB (200 mg) glass cartridges (Waters Corporation, Milford, MA), following a previously described protocol^51^. Briefly, cartridges were sequentially pre-conditioned, and 400 ml of filtered samples were loaded onto the cartridge at a flow rate of 5–6 ml/minute under continuous vacuum application. Analytes were consequently eluted from the cartridge with 100% methanol and concentrated by evaporation. Samples were then reconstituted in DMSO, diluted in growth media, added to cells at 200X concentration and incubated at 37 °C for up to 3 h. The final DMSO concentration was 0.5%.

**Table 1:** Day of the samples collection and location of the tested sites.

Sample	Collection date	Lat	Long
M2-1	8/8/18	38.052	− 77.348
M2-2	8/8/18	38.0412	− 77.381
M2-3	8/8/18	38.0172	− 77.38
M2-4	8/13/18	38.0099	− 77.384
M2-5	8/13/18	38.0024	− 77.381
M2-6	8/14/18	37.9818	− 77.366
M2-7	8/14/18	37.9645	− 77.342

### Generation of the GFP-GR-PR chimeric receptors

The GFP-GR-PR chimera containing GFP tagged N-terminal human glucocorticoid receptor (aa2-552) fused to the hPR-B LBD (621-end) was prepared in spectinomycin resistant GATEway (Thermo Fisher Scientific, Waltham, MA) Entry clone. As PR-A and PR-B differ only in their N-termini, the resulting construct represents both isoforms. The GFP-GR-PR constructs were sequenced throughout the entire cloned regions and were found to completely match the expected DNA sequence. Ampicillin resistant pFUGW lentivirus expressing clone was made with hygromycin selection marker and a Tet-regulated pTRE-tight promoter. Transfection-ready DNA was prepared using the Sigma GenElute XP Maxiprep kit and verified by agarose gel electrophoresis and restriction digest.

### Cell line generation and maintenance

The Hepa1 Tet-off cell line was generated using the hepatoma cell line Hepa-1clc7 that lacks aryl hydrocarbon receptor expression, tao BpRc1 (ATCC CRL-2218). Cells were transfected with the pTet-tTAk (Invitrogen) plasmid that expresses the tTA TetR-VP16 fusion from a minimal promoter downstream of 7 copies of the tet-O sequence thereby generating a tet-regulated version of tTA originally described by Shockett et al^52^. Since this plasmid contains no mammalian selectable antibiotic resistance gene, it was cotransfected with pSV2-neo (ATCC 37,149) at a one tenth molar ratio to pTet-tTAK as described by Walker et al^53^.,using Lipofectamine 2000 (Invitrogen). A polyclonal population was selected using 600 ug/ml G418 in media containing 5 ug/ml of tretracycline to minimize tTA expression, and single cell clones were screened using a Tet repressor antibody (TET01, Mo Bi Tec) after growth for 48 h in Tet Free FBS (Takara/ Clontech) The Hepa1 Tet-off cells were maintained in DMEM media supplemented with 10% FBS, 2 mM glutamine, and penicillin/streptomycin. For the development of stable cell line, the cells were first infected with lentivirus containing the GFP-GR-PR ampicillin resistant pFUGW lentiviral expression clone with a tet-regulated pTRE-Tight promoter and later challenged with hygromycin to generate a polyclonal cell line. The cells were subsequently infected with lentivirus containing the mCherry-NF1 ampicillin resistant pFUGW lentiviral expression clone with a tet-regulated pTRE-Tight promoter and later challenged with zeocin to generate a polyclonal cell line. Single cell clones were generated by limiting dilution and tested for GFP-GR-PR and mCherry-NF1 expression levels and response to P4 treatment.

### Cell treatment and preparation for high-throughput imaging

Prior to imaging, cells were plated in 384-well plates (Matrical, Catalog Number MGB101-1-2-LG-L) at a density of 10,000 cells per well in DMEM medium containing 10% charcoal stripped serum (Hyclone, Logan, UT) without tetracycline to allow the expression of the GFP-GR-PR and mCherry-NF1. Cells were treated with DMSO or Ethanol (vehicle controls), or various concentrations of P4, Gestogene, Norgestimate, Altrenogest, Chlormadinone Acetate, Norethindrone, Norgestrel, Levonorgestrel, Etonogestrel, Asoprisnil, Ulipristal Acetate, RU-486, Dex, E2, Testo and HydroCort for 2 h, unless specified otherwise. For the time-response experiment cells were treated with 100 nM P4, RU-486, Dex, E2, and HydroGort for various lengths of time and either imaged by time-laps microscopy or fixed upon treatment for later imaging. Water sample extracts as well as blank samples (distilled water subjected to the same extraction procedure) were applied for 2 h at 37 °C at a final concentration of 200X, if not specified otherwise. All water samples as well as the tested compounds were each screened in at least 4 individual wells. After treatment, cells were fixed with 4% paraformaldehyde in PBS for 10 min and washed 3X with PBS. Cells were further stained with DAPI (4′,6-diamidino-2-phenylindole, SC-3598, ChemCruz) dissolved in PBS (5 µg/ml) for 10 min, washed 3X with PBS, and either imaged immediately or kept in PBS at 4 °C for later imaging.

### Imaging and analysis

A Yokogawa CV7000S high-throughput dual spinning disk confocal microscope was used for fully automated collection of images. Images were acquired with a 40X Olympus PlanApoChromat air objective (NA 0.9) and two sCMOS cameras (2560 X 2160 pixels) using camera binning of 2X2 (Pixel size 325 nm). A single imaging plane was used and the 2X2 binning allowed us to reduce the size of the files and streamline the careening process. Samples were imaged using 405 nm, 488 nm, 561 nm excitation lasers and a fixed 405/488/561/640 nm dichroic mirror on the excitation side. On the emission side, the emitted light was split by a fixed 561 nm emission dichroic mirror. Specifically, emitted light with wavelengths below 561 nm was reflected by the emission dichroic mirror at a 90-degree angle to the camera switchable 445/45 or 525/50 nm emission bandpass filters mounted on a filterwheel (Camera #1). Emitted light with wavelengths above 561 nm passed through the mirror and to the other camera with switchable 600/37 or 676/29 nm emission bandpass filters mounted on another filter wheel (Camera #2). Camera #1 and Camera #2 were positioned perpendicular to each other. DAPI (Ex. 405 nm—Em 445/45 nm) and GFP (Ex. 488 nm—Em. 525/50 nm) were always acquired in separate exposures. Additional images were taken on PerkinElmer (Waltham, MA) Opera QEHS High-Content Screening platform. This system employed a 40X water immersion objective lens, laser illuminated dual Nipkow spinning disk, and cooled charge-coupled device cameras to digitally capture high-resolution confocal fluorescence micrographs (323 nm pixel size with 2X2 camera pixel binning). On average, 8 fields per well were imaged using a single imaging plane.

An image analysis pipeline was customized using the Columbus software (PerkinElmer) to automatically segment the nucleus using the DAPI or the mCherry channel and then construct a ring region (cytoplasm) around the nucleus mask for each cell in the digital micrographs. The algorithm also measured the mean GFP-GR-PR intensity in both compartments, and translocation was calculated as a ratio of these intensities. The values from 4 independent wells per sample (each representing the average of 8 imaging fields per well) were further normalized to the value for the respective solvent control (DMSO for the water samples or EtOH for the tested chemical compounds). Finally, an average normalized translocation value was calculated for each sample.

### Calculation of P4 equivalent (P4-EQ) activities in the water samples

After the generation of a calibration dose–response curve for the GFP-GR-PR translocation in response to 0 nM, 0.25 nM, 0.5 nM, and 1 nM of P4, a linear fit was performed yielding a formula y = 1.028x + 1 (R^2^ = 0.9888). To calculate P4-EQ for a given sample, its translocation value (y) was used using the formula x = (y − 1)/1.028, where x is the P4-EQ in nM.

### Statistical analyses of the data

Data were analyzed using the graphical and statistical functions of SigmaPlot 14 (SPSS Inc., Chicago, IL). From the repeated experiments (4 or more replicas) upon the normalization, the mean value ± s.e.m and z′ (z-score) were calculated for each sample. The mean values were used in a one-way analysis of variance test. If a significant F-value of *P* < 0.05 was obtained, a Holm-Sidak's multiple comparison versus the control group analysis was conducted.

## Results

### Generation of a cell line expressing a chimeric receptor responsive to P4

**Figure 1:**
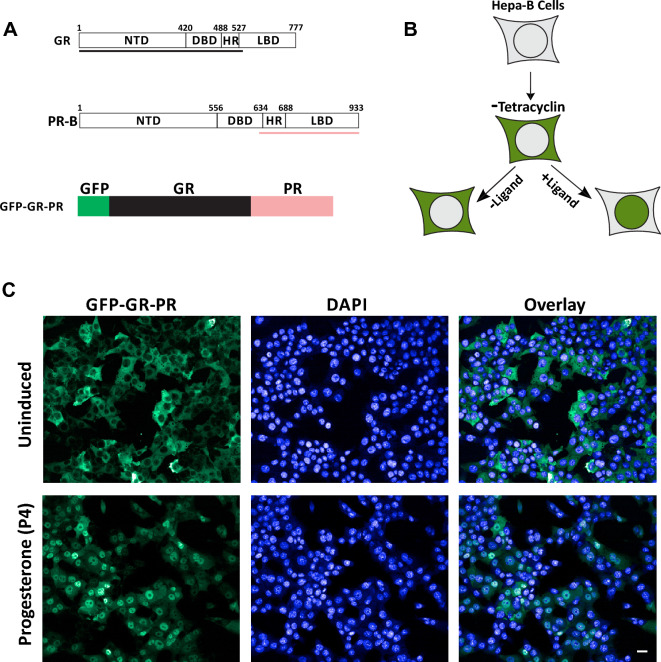
Establishment of a cell line expressing GFP-GR-PR chimeric receptor under Tetracycline regulation. (**A** and **B**) Schematics of the strategy used for generating GFP-GR-PR chimera and of the GFP-GR-PR translocation in response to hormonal treatment. (**C**) GFP-GR-PR translocation in a mammalian cell line (Hepa-B) upon stimulation with 100 nM Progesterone (P4) for 2 h as detected by the CV7000 automated imaging analysis system. Nuclei are stained with DAPI. Scale bar, 20 μm.

The GFP-GR-PR chimera containing a GFP reporter and human glucocorticoid receptor N-terminal fragment (amino acids (aa) 2-552) fused to PR-B (621 aa-end) fragment containing the PR ligand binding domain (LBD) was constructed in lentiviral vectors (Fig. 1A). The longer (PR-B) isoform was used as a reference in the Fig. 1A schematics; however, the PR-B fragment sequence is also present in the PR-A, because the two isoforms differ only by a 165 amino acid extension at the N-terminus of the PR-B isoform. Therefore, the chimeric receptor represents both PR isoforms and was further introduced into Hepa1 tetracycline (Tet)-off cells to establish a stable cell line in which the GFP-GR-PR construct translocated to the nucleus upon treatment with 100 nM P4 (depicted schematically in Fig. 1B and in the microphotographs in Fig. 1C). Thus, we generated a mammalian cell line expressing GFP-GR-PR upon removal of Tet from the growth media and the GFP-GR-PR translocates from the cytoplasm to the nucleus following exposure to P4.

### Time- and concentration-dependent response of the GFP-GR-PR chimera to P4

**Figure 2:**
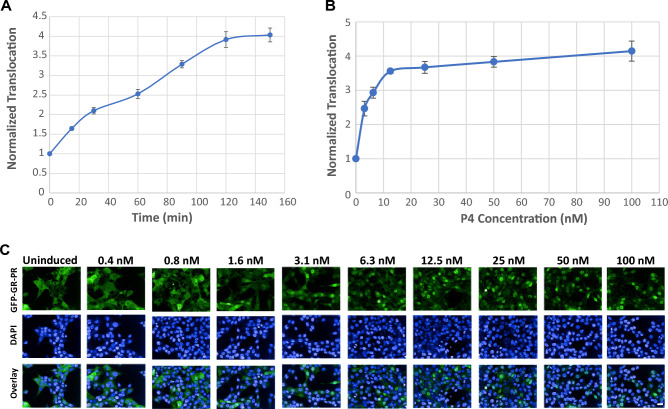
Establishment of the time- and concentration-dependent response of the GFP-GR-PR chimera to P4. (**A**) Time-dependent GFP-GR-PR translocation upon treatment with 100 nM P4. Each value was normalized to the control (EtOH vehicle treated) sample. Error bars represent the mean value ± s.e.m, n = 4. (**B**) GFP-GR-PR translocation in response to varying concentrations of P4 over 2 h incubation. The EC_50_ = 3 nM and the Z’ for the assay varied between 0.5 and 0.8. Error bars represent the mean value ± s.e.m, n = 4. (**C**) Representative images of the GFP-GR-PR nuclear translocation upon treatment with different concentrations of P4 for 2 h as detected by the CV7000 automated imaging analysis system. Nuclei are stained with DAPI. Scale bar, 20 μm.

First, to establish the optimal time for treatment, we exposed cells to 100 nM P4, fixed them with 4%PFA at different times up to 3 h, and compared the level of the GFP-GR-PR translocation to the control (unstimulated, EtOH-treated) cells. To calculate translocation efficiency of the GFP-GR-PR we implemented an algorithm for cytoplasm and nuclear segmentation^54,55^ and found that the GFP-GR-PR translocation was time-dependent (Fig. 2A). Even though significant translocation was detected upon 15 min of incubation with P4, the response at 2 h and 3 h was much higher, showing a fourfold increase. We concluded that 2 h with P4 treatment was sufficient for the GFP-GR-PR translocation.

Next, to determine the sensitivity of the assay, we treated cells with different concentrations of P4 for 2 h and observed a concentration-dependent increase of the GFP-GR-PR translocation that plateaued around 50 nM P4 (Fig. 2B, see also representative micrographs in Fig. 2C). We concluded that the rate of translocation of the GFP-GR-PR chimers from the cytoplasm to the nucleus can be used as a read-out to its response to PR agonists.

### GFP-GR-PR chimera also translocates in response to an antagonist

**Figure 3:**
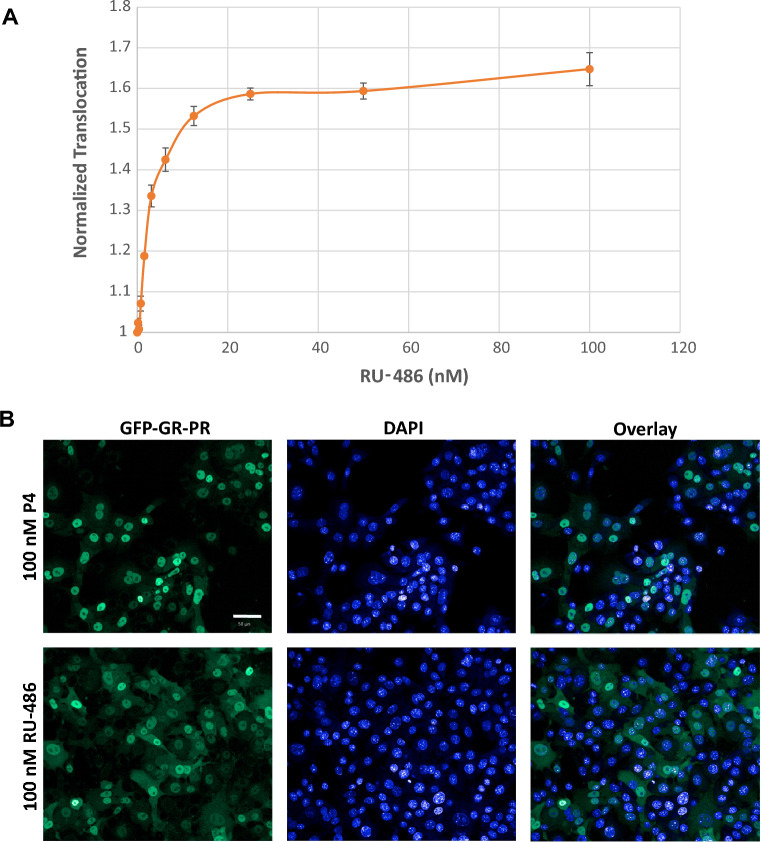
Nuclear translocation of GFP-GR-PR in response antagonist RU-486 (Mifepristone). (**A**) RU-486 induced significant nuclear translocation of the GFP-GR-PR chimeric receptor upon 2 h incubation at 37 °C. The EC_50_ = 3 nM and the Z′ for the assay varied between 0.5 and 0.7. (**B**) Representative images of the GFP-GR-PR translocation in response to varying concentrations of RU-486 after 2 h of incubation at 37 °C as detected by the CV7000 automated imaging analysis system. Nuclei are stained with DAPI. Scale bar, 50 μm.

After we established the GFP-GR-PR chimera time- and concentration-dependent response to a known PR agonist (P4), we determined its translocation response to a known PR antagonist, RU-486 (Mifepristone). To achieve this, we incubated the GFP-GR-PR-expressing cells with increasing concentrations of RU-486 for 2 h at 37 °C and observed concentration-dependent increase of the GFP-GR-PR nuclear intensity (Fig. 3A). However, the GFP-GR-PR translocation was partial even in the presence of 100 nM RU-486 (Fig. 3B) and plateaued at translocation level corresponding to 6 nM P4. We concluded that in addition to detecting PR agonists our assay can also detect PR antagonists. This feature of the assay allows detection of both agonists and antagonists and thus does not require testing in “antagonists” mode (in the presence of saturating P4). However, additional assays will be required, if the goal of the screens is to distinguish between these two modes of PR translocation.

### Real-time and in vivo measurement of translocation of the GFP-GR-PR chimera

**Figure 4:**
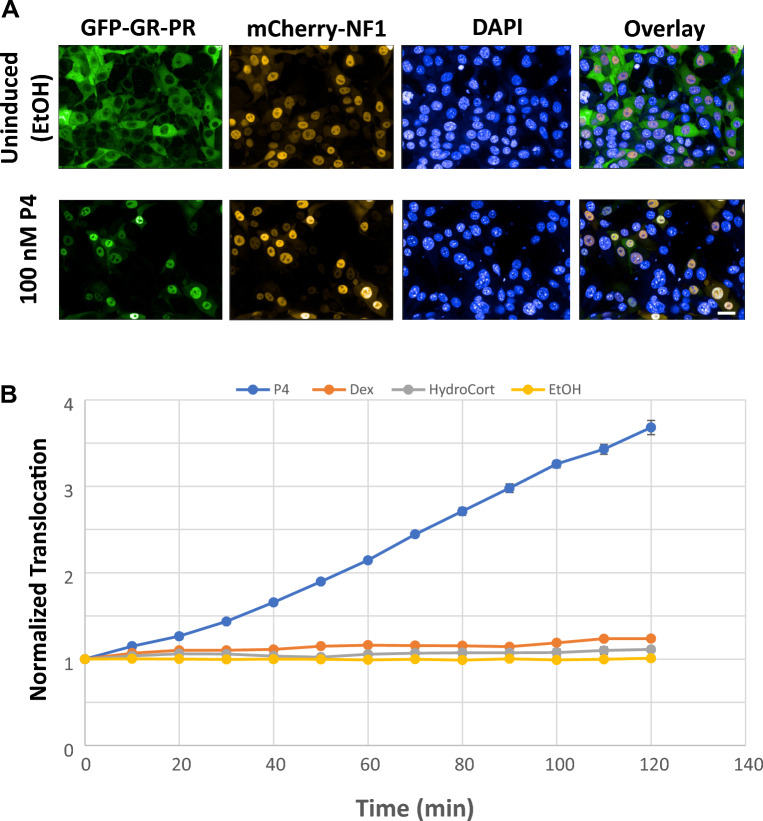
Expression of a nuclear marker (mCherry-tagged NF1) enables the real-time live-cell imaging of the GFP-GR-PR translocation. (**A**) Images of cells expressing the GFP-GR-PR and mCherry-NF1 proteins after exposure to the vehicle (EtOH) or 100 nM P4 for 2 h at 37 °C, PFA fixation and DAPI nuclear staining. Images were collected by the Opera QEHS High-Content Screening platform. Scale bar, 20 μm. (**B**) GFP-GR-PR translocation in response to 100 nM of P4, Dexamethasone (Dex), Hydrocortisone (HydroCort), and Vehicle (EtOH) control as determined by the live cell 10 min-time-lapses for 2 h at 37 °C performed on the CV7000 automated imaging analysis system. The mCherry-NF1 intensity in the nuclei was used for nuclear segmentation. Error bars represent the mean value ± s.e.m, n = 4.

**Figure 5:**
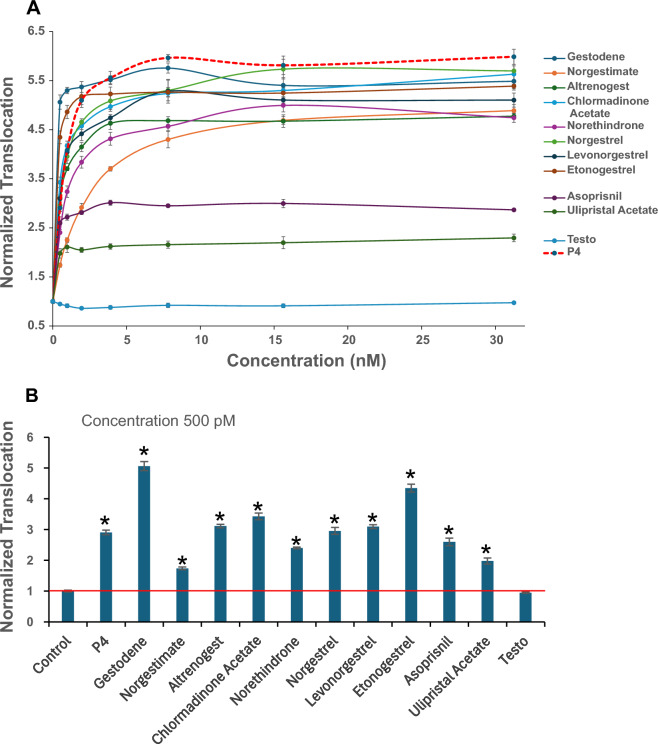
Real-time live-cell imaging of the GFP-GR-PR translocation in response to progestogens. (**A**) GFP-GR-PR translocation in response to varying concentrations of PR synthetic agonists, PR modulators as well as the AR specific ligand testosterone (Testo) upon 2 h treatment. Negative (solvent) control was used to normalize the translocation response to each ligand. Progesterone (P4) was used as positive control. The Z′ for the assay varied between 0.5 and 0.85. Error bars represent the mean value ± s.e.m, n = 4. (**B**) GFP-GR-PR translocation in response to 500 pM of the selected ligands. Error bars represent the mean value ± s.e.m, n = 4 (*P* < 0.05, asterisks).

To further develop our system and to adapt it for detection of the GFP-GR-PR translocation in real-time in live cells, we need a reliable marker that allows unambiguous detection and segmentation of the nuclei. In the PFA-fixed cells, the nuclear DNA stain DAPI serves this role; however, in live cells expressing the GFP-GR-PR, the nuclear boundaries are not easily discernable and preclude the application of the nucleus segmentation algorithm on which translocation measurements are based. To overcome this hurdle, we introduced a constitutive nuclear marker (Nuclear Factor1) tagged with the red fluorescent protein mCherry (mCherry-NF1) also under Tet-off regulation. The cells expressing this marker had clearly distinguishable nuclei in the mCherry channel (Fig. 4A). Our live cell time-laps illustrated real time translocation of the GFP-GR-PR in response to 100 nM of P4 (Supplemental Movies 1A and 1B), and 100 nM RU-486 (Supplemental Movies 2A and 2B) whereas GFP-GR-PR localization was unchanged when vehicle (EtOH) treatment was applied (Supplemental Movies 3A and 3B). Moreover, the presence of mCherry-NF1 in the cell nuclei used for nuclear segmentation throughout the live cell time-laps experiments allowed the measure of time-dependent GFP-GR-PR translocation in response to various treatments. The response to 100 nM of P4, Dex, HydroCort, and Vehicle (EtOH) control is shown in Fig. 4B. The time-response curve generated from these time-lapses in live cells is similar to the one generated using fixed cells (Fig. 2A) demonstrating that mCherry-NF1 fluorescence can be successfully used for nuclear segmentation. Interestingly, even though the levels of translocation to Dex were very low, they were not unsignificant (Supplemental Fig. 1), raising the question on specificity of GFP-GR-PR chimera. The hGR fragment used in the GFP-GR-PR construct was used previously for the development of GFP-GR-RAR, GFP-GR-ER and GFP-GR-TR chimeric reporter cell lines^55–57^ and these chimeras never showed any cross reactivity with GR ligands (because the GR N-terminal fragment (2–552 aa) fragment lacks the GR-specific LBD). Considering this, an alternative explanation is that PR LBD itself may have a low but discernable affinity for Dex. In support to this notion, it was shown previously that some synthetic steroids with androgenic, antiandrogenic, estrogenic and glucocorticoid activities also exert progesterone-like activity^21,22,58^. Moreover, many of the progestins used in endocrine therapy show off-target glucocorticoid activity^59^. To further demonstrate that the GFP-GR-PR chimera can specifically detect PR ligands, we treated the cells with various concentrations of known synthetic progestogens with agonistic activity used in human contraception or in veterinary hormone therapy (Gestogene, Norgestimate, Chlormadinone Acetate, Norethindrone, Norgestrel, Levonorgestrel, Etonogestrel and Altrenogest, respectively)^60,61^. As shown in Fig. 5A, all tested agonists induced concentration-dependent translocation of the chimera. Considering that some progestogens are known to also interact with AR^62^ we wondered whether the AR specific ligand testosterone (Testo) could induce GFP-GR-PR translocation. As shown in Fig. 5A, the GFP-GR-PR chimera did not translocate in response to any of the tested concentrations of testosterone. Next, we tested the effects of two PR modulators (Asoprisnil and Ulipristal Acetate)^63^ on the GFP-GR-PR translocation and demonstrated that they also interact with the chimera and induced its partial translocation (Fig. 5A). Finally, even at 500 pM concentration all tested PR ligands induced statistically significant increase in the GFP-GR-PR translocation (Fig. 5B). We concluded that GFP-GR-PR chimera specifically detects PR ligands as well as PR modulators.

Our experiments also demonstrate that the GFP-GR-PR cell line expressing mCherry-NF1 protein can be used successfully in real-time translocation studies, potentially eliminating the PFA fixation and nuclear staining steps. This streamlines the screening process and reduces the time, cost, and the toxicological impact of this screening method.

### Screening of river water samples for PR-interacting contaminants

**Figure 6:**
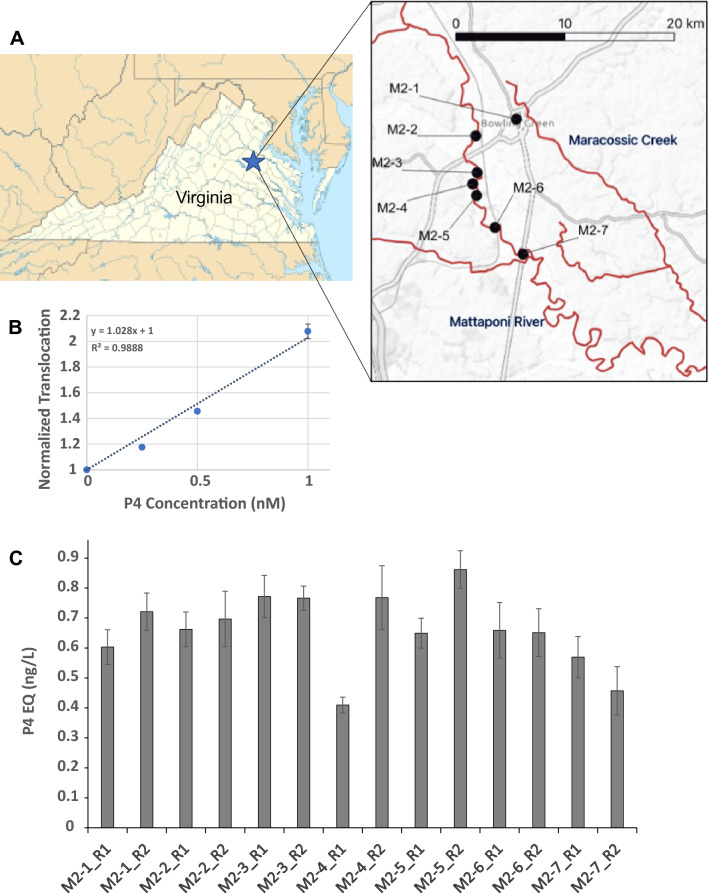
Geographic locations of the collection sites of the river water samples and screening results**.** (**A**) Map of the river water collection sites. (**B**) A standard curve for the GFP-GR-PR translocation upon treatments with known concentrations of P4 was used to recalculate the P4 equivalent (P4-EQ) activities in the water samples (presented as ng/L). Error bars represent the mean value ± s.e.m, n = 4. Using the Holm-Sidak’s multiple comparison versus the control group analysis all samples were significantly different from the blank (DMSO) control (*P* < 0.05).

Next, we used the GFP-GR-PR expressing cell line to test concentrated water samples, collected at the sites indicated in Fig. 6A, from the Mattaponi River in Virginia. We discovered a low but reproducible activity in all highly (200X) concentrated samples (Supplemental Fig. 2), suggesting contamination of these sites with chemicals interacting with PR.

### P4 equivalent (P4-EQ) activities in the river water samples

Biological activity present in the collected samples was further compared to the translocation activity elicited by known levels of P4. To do that we generated a P4/translocation dose–response curve (up to 1 nM P4) which encompasses just the linear portion of the P4/translocation dose–response (Fig. 6B). This linear dose–response curve was used as a standard to calculate the P4 equivalent (P4-EQ) activities of the concentrated samples (see Material and Methods section) which was then recalculated to the original undiluted sample. We concluded that river water samples contained progesterone receptor interacting contaminants ranging from 0.3 to 0.86 ng/L (0.001 to 0.003 nM) P4-EQ (Fig. 6C).

## Discussion

Dysregulation of sex-steroid receptor functions, including the PR, has been implicated in numerous pathological conditions including cancer, obesity, neuroendocrine disorders, cardiovascular disease, hyperlipidemia, infertility, and other reproductive disorders [reviewed in^6,64^]. Serious ecotoxicological consequences associated with exposure to progestins are also emerging. Exposure to these chemicals induces transcriptional changes in zebrafish^65–67^. Progestins at pM concentrations impact sex development and reproduction in fish^68–72^. Finally, Brockmeier et al^73^ also demonstrated that exposure of Eastern Mosquitofish to paper mill effluent at a site in North Central Florida elicited gene expression alterations consistent with progesterone and androgen exposure.

Current screening methods and cell lines for detection of EDCs have significant limitations^74–76^. Rapid, sensitive, specific, inexpensive and high-throughput effect-based methods for EDC detection are required.

We previously described a translocation-based screening method for detection of biologically active compounds interacting with multiple NRs in mammalian cells^77,78^. We amid to develop a similar type of screening approach to detect both PRA- and PRB-interacting contaminants. Even though, PRA and PRB have the same LBD, the GFP-tagged PRB is mostly cytoplasmic at uninduced conditions while the GFP-PRA is largely nuclear^79^. To create a biosensor that will report on the activation of both isoforms by known and novel PR ligands, we created GFP fusion of the GR N terminus and PR LBD (Fig. 1A). The GFP-GR-PR chimeric receptor is cytoplasmic before induction and translocates to the nucleus in response to a ligand (Fig. 1B, C). We observed time- and concentration-dependent increase in GFP-GR-PR translocation in response to P4 (Fig. 2A–C). A PR antagonist RU-486 (Mifepristone), widely used in pregnancy prevention pills, also induced GFP-GR-PR translocation in a concentration-dependent manner, albeit plateauing at lower translocation level than P4 treatment (Fig. 3A–C), thus suggesting that the chimera could detect both types of ligands (agonists and antagonists) in chemical libraries and in environmental samples.

We developed an additional modification of the assay that permits real time and in vivo screening of samples for PR-interacting EDCs by introducing a nuclear marker (mCherry-NF1) into the GFP-GR-PR-expressing cells. This real-time in vivo monitoring (Fig. 4A, see also Supplemental Movies 1 to 3) eliminates the need for fixation and nuclear (DAPI) staining of the cells, which shortens screening time and reduces potential toxicological impact of the procedure. This assay requires an environmental chamber which is not a standard adaptor in high-throughput screening systems. Thus, cell fixation is optional and even if cell fixation is performed, this assay improves processing time by eliminating the need for nuclear staining. We used this in vivo setup to test the GFP-GR-PR concentration-dependent translocation in response to a battery of PR ligands and modulators (Fig. 5A, B) and demonstrated the ability of the chimera to recognize these PR ligands, while it did not translocate in response to testosterone, an AR ligand.

Next, we tested GFP-GR-PR cells for PR-interacting contaminants in environmental water samples, collected from Mattaponi River (Virginia) in 2018^78^. All samples induced partial GFP-GR-PR translocation indicative of a low, but widely disseminated contamination of river water samples with PR-interacting contaminants (Fig. 6).

Finally, we recalculated the P4 equivalent (P4-EQ) activities of river water samples. The highest P4-EQ was 0.86 ng/L (0.003 nM). Considering that this concentration is in the lower range of that previously reported in surface waters [between a few to tens ng/L (reviewed in^21^], the overall biological impacts of the detected activities may be negligible. However, P4-EQ values, even though informative, should be interpreted with caution. For example, treatment with 25–100 nM of RU-486 (Fig. 3A) results in a “Normalized Translocation” value of ~ 1.7, which corresponds to a much lower (~ 0.7 nM) P4-EQ. Therefore, low translocation values may indicate that some samples could contain relatively high levels of low translocation-potency PR ligands, such as RU-486 or the PR modulators Asoprisnil and Ulipristal Acetate (see Figs. 3 and 5, respectively).

## Conclusions

We developed a novel assay for rapid high-throughput screening of PR-interacting chemicals. The use of living cells eliminates the need for cell fixation and nuclear staining. The assay facilitates search for novel PR ligands in chemical libraries and aids in detection and monitoring of PR-disrupting activities in environmental samples.

## Supplementary Information

Supplementary Video 1.

Supplementary Video 2.

Supplementary Video 3.

Supplementary Video 4.

Supplementary Video 5.

Supplementary Video 6.

Supplementary Information.

## Data Availability

The datasets generated during and/or analyzed during the current study are available from the corresponding authors on reasonable request.

## Abbreviations

PR: Progesterone receptor
GR: Glucocorticoid receptor
P4: Progesterone
RU-486: Mifepristone
DMSO: Dimethyl sulfoxide
Tet: Tetracycline
Dex: Dexamethasone
HydroCort: Hydrocortisone
LBD: Ligand binding domain
