# Supplementary material for: Novel biosensor for high-throughput detection of progesterone receptor-interacting endocrine disruptors
Source: Sci Rep. 2024 Mar 6;14:5567. doi: 10.1038/s41598-024-55254-8 (PMC10917811; doi:10.1038/s41598-024-55254-8)

Supplemental Information

Novel Biosensor for High-Throughput Detection of Progesterone Receptor-Interacting Endocrine Disruptors

Diana A. Stavreva[1*], Lyuba Varticovski[1], Razi Raziuddin[1], Gianluca Pegoraro[1], R. Louis Schiltz[1] and Gordon L. Hager[1*]

[1] Laboratory of Receptor Biology and Gene Expression, 41 Medlars Dr., National Cancer Institute, NIH, Bethesda, MD 20892-5055

*Corresponding Authors:

hagerg@exchange.nih.gov, phone: 240-760-6618, fax: 301-496-4951

stavrevd@mail.nih.gov, phone: 240-760-6722, fax: 301-496-4951

Supplemental Figure Captions:

Supplemental Figure 1. Low but significant GFP-GR-PR nuclear translocation in response to Dexamethasone (Dex) treatment. Data are derived from the live cell 10min-time-lapses for 2h at 37°C performed on the CV7000 automated imaging analysis system. Error bars represent the mean value ± s.e.m, n=5 (P<0.05, using the 0h EtOH treatment as a control). Asterisks are for the Dex datapoints.

Supplemental Figure 2. Screening of water samples for GFP-GR-TR nuclear translocation. (A) GFP-GR-PR nuclear translocation in response to treatment with 200X concentrated water samples collected in duplicates (gray bars and the blue bar) and to different concentrations to the PR agonist, P4 (red bars). Error bars represent the mean value ± s.e.m, n=4 (P<0.05, asterisks). (B) Representative images of the GFP-GR-PR translocation upon stimulation with vehicle (0.5%

DMSO), 200x concentrated sample M2-3_R1 (blue bar in A), and 0.5 nM P4 for 2h at t 37°C as detected by the CV7000 automated imaging analysis system. Scale bar, 50 μm.

Supplemental Movie 1. Complete GFP-GR-PR translocation in the presence of 100nM Progesterone. A) A time lapse where images of the GFP-GR-PR and the mCherry-NF1 fluorescence were recorded at 10-minutes intervals for the total of 2h. B) Same time laps showing only the GFP-GR-PR fluorescence.

Supplemental Movie 2. Incomplete GFP-GR-PR translocation in the presence of 100nM Mifepristone (RU-486). A) A time lapse where images of the GFP-GR-PR and the mCherry-NF1 fluorescence were recorded at 10-minutes intervals for the total of 2h. B) Same time laps showing only the GFP-GR-PR fluorescence.

Supplemental Movie 3. Vehicle (0.1% ethanol) treatment does not affect the GFP-GR-PR localization. A) A time lapse where images of the GFP-GR-PR and the mCherry-NF1 fluorescence were recorded at 10-minutes intervals for the total of 2h. B) Same time laps showing only the GFP-GR-PR fluorescence.

Supplemental Figure 1

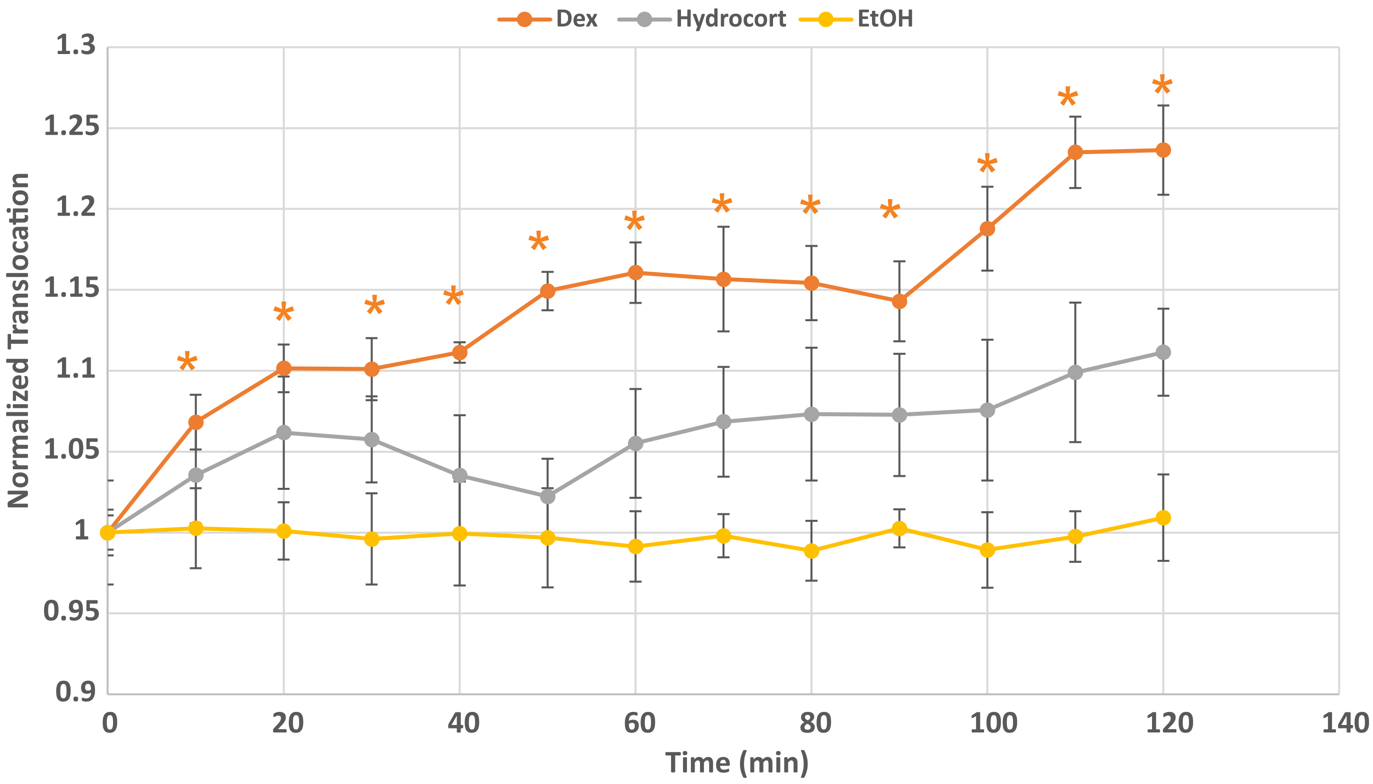

Supplemental Figure 2

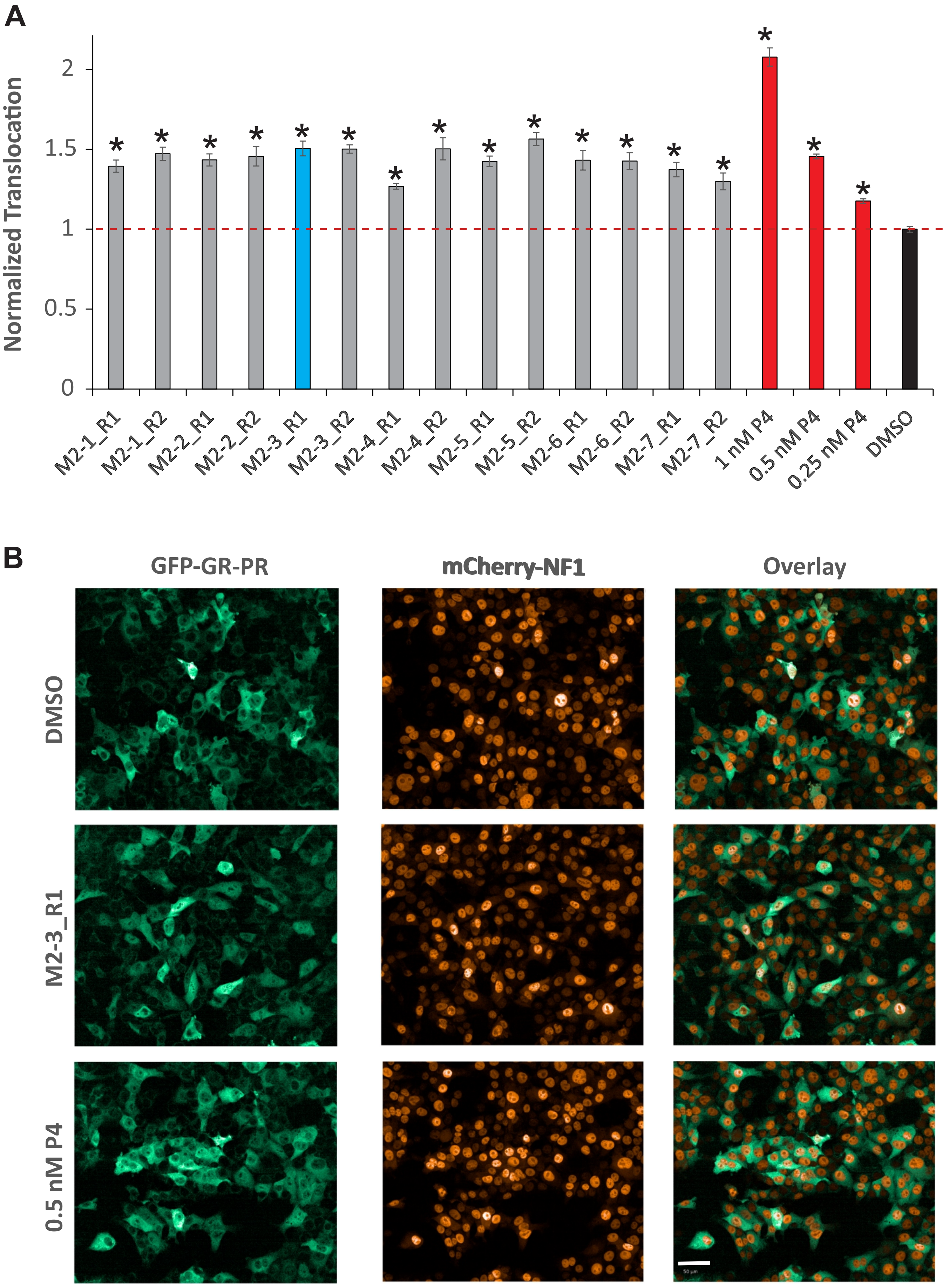

Supplement: Supplementary file 7 — Supplementary Information. [file 41598_2024_55254_MOESM7_ESM.pdf]
